# A review of the global use of fishmeal and fish oil and the Fish In:Fish Out metric

**DOI:** 10.1126/sciadv.adn5650

**Published:** 2024-10-16

**Authors:** Patricia Majluf, Kathryn Matthews, Daniel Pauly, Daniel J. Skerritt, Maria Lourdes D. Palomares

**Affiliations:** ^1^ Oceana, 1025 Connecticut Ave. NW, Suite 200, Washington, DC 20036, USA.; ^2^ Institute for the Oceans and Fisheries, University of British Columbia, 2202 Main Mall, Vancouver, BC V6T 1Z4, Canada.

## Abstract

Aquacultured carnivorous species consume most of the world’s fishmeal and fish oil (FMFO), which itself is primarily derived from small pelagic fish. This has raised concerns about the practice’s impact on wild fish stocks, ecosystems, and coastal communities that rely on these fish. The aquaculture industry claims a decreasing dependence on wild fish, relying on the Fish In:Fish Out (FIFO) metric as a ratio of the quantity of wild fish required for farmed fish production. This is misleading because it usually assumes constant FM or FO yields, inclusion rates and feed conversion ratios, which vary widely. Thus, a constant FIFO value for a given species cannot be assumed. Furthermore, low FIFO values resulting from averaging carnivores and herbivores conceal the high feed requirements of carnivore species. The increasing use of FMFO from by-products does not demonstrate a decreased use of wild fish but rather reflects a growing demand for FMFO, particularly for the fast growing and valuable salmon and shrimp farming industries.

## Teaser

Aquaculture’s wild fish dependence raises concerns, but low or no fish-oil feeds may enhance resilience and sustainability.

## INTRODUCTION

The farming of carnivorous fish and invertebrates, i.e., “fed aquaculture,” is using an increasingly large share of the global fishmeal and fish oil (FMFO) production (*1*–*4*), which is often manufactured by “reducing” some of the most nutrient-rich wild fish in the world, i.e., small pelagic fish, such as anchovies and sardines, into feed for farmed animals. Reduction fisheries compete with other marine predators, such as seabirds, marine mammals, and carnivorous fish, affecting the productivity and resilience of some of the most productive ecosystems in the world (*5*, *6*). Moreover, they often operate regions with poor coastal communities that rely on access to these fish for sustenance and livelihoods (*7*–*9*), which has raised concerns about the global impact of FMFO production and the outlook for the aquaculture industry (*9*–*13*).

Discussion of those impacts often references the Fish In:Fish Out (FIFO) metric, the standard ratio used to quantify how much wild fish is used to produce farmed fish and is often used as an indicator of the impact of aquaculture on wild fish stocks (*11*, *14*–*21*). Since Naylor *et al.* (*11*) first estimated that it can take up to 5 kg of wild fish to produce 1 kg of farmed carnivorous fish, controversy has arisen around the calculation and meaning of FIFO (*11*, *14*, *16*–*20*). The reduction industry itself, i.e., those organizations that “reduce” small pelagic fish to FMFO products, largely represented by IFFO (originally the International Fishmeal & Fish Oil Organization, now the Marine Ingredients Organization), have tried to demonstrate a decreasing dependence of aquaculture on wild fish and argue that it is not imposing, but instead, relieving pressure on wild fish stocks (*15*, *21*–*28*).

However, in a recent review of the sustainability of aquaculture feed, Tacon *et al.* (*16*), going back to the origin of FIFO, explained “that the FIFO ratio was never intended to be a precise measurement of how much wild fish is required to produce a given amount of farmed fish. The metric itself was to bring attention to the reliance of the aquaculture feed industry on wild capture fisheries. Further with much of the aquaculture sector seeking to portray farmed seafood as a solution or alternative to wild capture fisheries, the FIFO ratio highlighted the specific dependence aquaculture has on wild capture fisheries.”

Here, we explore the factors that shape the current use of wild-caught fish by the aquaculture industry through the “lens” provided by the FIFO metric. We first examine the component variables of FIFO and their drivers. Next, we look at the reduction fisheries and the increasing use of by-products of the seafood processing industry to produce FMFO that meets the growing demand for these commodities, particularly for FO. Last, by describing the current uses of FM and FO by the aquaculture industry, we demonstrate how most of this global supply, especially of FO, now being used by the aquaculture industry purportedly to “contribute to global food security” (*2*) is, in fact, primarily being used to produce high-value, globally traded seafood that benefits only the few who can afford it.

## THE FIFO METRIC

The FIFO metric was first formulated by Tacon and Metian (*17*) to estimate the amount of fish from capture fisheries required to produce a unit of farmed fish. FIFO is calculated separately for fish oil and fish meal according to Eq. 1

$${\text{FIFO}}_{\text{FM or FO}}=\frac{\text{FM or FO inclusion rate}}{\text{FM or FO yield}}\times \text{FCR}$$ (1)

where the FM or FO inclusion rate is the amount of fishmeal or fish oil included in the feed; FM or FO yield is the amount of fishmeal or fish oil obtained from a unit of raw fish through the reduction process; and FCR is the feed conversion ratio, or the amount of feed (e.g., in kilograms) required to produce (1 kg of) farmed fish.

The following example for 1 kg of carnivorous fish like salmon (Table 1), with yields for FM = 22.5% and for FO = 5%, inclusion rates for FM = 24% and for FO = 16%, and an FCR = 1.25%, shows that although there is less FO than FM in the feed, more fish is needed to produce the FO, and the resulting FIFO shows that at least 4 kg of wild fish are needed to produce 1 kg of farmed fish.

**Table 1.** An example calculation of the Fish In:Fish Out (FIFO) metric or the amount of fish needed to produce 1 kg of carnivorous fish like salmon. FIFO for FM and FO are calculated separately using the following values: FMyield = 22.5%, FOyield = 5%, inclusion rates for FM = 24% and for FO = 16% and FCR = 1.25%.

	FM	FO
Inclusion in feed	1.25 * 0.24 = 0.3 kg of FM	1.25 * 0.16 = 0.2 kg of FO
FIFO	24/22.5 * 1.25 = 1.33 kg	16/5 * 1.25 = 4.0 kg

### Rising costs decrease inclusion rates

Inclusion rates—the fraction of FMFO contained in compound feeds for aquaculture—have decreased, from 23 to 8% over the past two decades (*29*). This was largely a result of increasing costs and decreasing supplies of FMFO, coupled with an increasing demand from the aquafeed industry (*2*, *3*, *30*–*32*).

Aquaculture feeds with high inclusion rates are still used, but more strategically, at critical stages of the life cycle of the farmed fish (*33*), with less valuable FM made from by-products feeding lower-valued freshwater species for local consumption. This approach helps to reduce the reliance on FMFO from wild fish (*11*). Replacing FO with plant-based oils for freshwater fish is easier than for marine and diadromous carnivorous species like Atlantic salmon (*Salmo salar*), which demand more FO (*34*).

If the production of aquafeeds continues to grow—having already tripled between 2000 and 2020 (*29*, *35*)—while the global supply of FMFO continues to decrease, even assuming a developing production of FMFO from by-products, then FMFO inclusion rates will likely continue to decline (*31*, *36*, *37*) even if the absolute amount of FMFO used by the aquaculture sector continues to grow.

### Use of global average FMFO yields hides large variability

FMFO are the final coproducts of fish reduction, with the pressed solids ground into fishmeal and the FO distilled from the remaining liquid. Generally speaking, regardless of the species or any other conditions, FM yields tend to be relatively constant, normally ranging between 16 and 25% by weight of the raw material (*38*–*44*). The reported average yield of 22.5% (*21*, *45*–*47*) is used as a standard by the reduction (www.IFFO.com) and aquaculture industries (*48*), though production improvements may have increased this figure to 23.5 to 24.5% (*20*, *21*, *43*).

Fish oil yields, conversely, are lower and more variable. The industry “average” of 5% (*21*, *45*–*47*) is derived from a huge range from 0.2 to 25% of values. This variability is driven by the variability of the fat content of the source fish, which is largely determined by their diet composition, which itself is influenced by environmental fluctuations affecting ocean primary productivity (*49*–*51*). Because fat content varies within and among species, seasonally, and with age, sex, location, and reproductive stage, the amount of fish needed to produce a given amount of fish oil can vary widely.

Catches of juvenile or temperature-stressed Peruvian anchoveta (*Engraulis ringens*) tend to deliver a lower FO yield than those of healthy adults, thus requiring higher catches to produce the same total amount of FO (*44*). Thus, because of the highly variable nature of the Peruvian upwelling ecosystem, the fat content and, consequently, the FO yield of anchoveta, the single species contributing most to FM and FO production (*2*, *52*) is highly variable. The anchoveta required to produce a tonne (tonne = 1000 kg) of FM ranges from 4 to 4.3 tonnes (yield: 22.7 to 25%), while for a tonne of FO, the range is much wider, with up to 55 tonnes of fish to produce 1 tonne of FO (yield 1.8 to 8%), depending on the year, season, and the size (age) of the fish (*44*). These values may further fluctuate on the basis of the frequent environmental changes in primary productivity typical of the Peru upwelling ecosystem (*51*). For this reason, using a constant value for FO yield, as the industry currently does [e.g., the Global Seafood Alliance Certification Standard (*53*)], can be misleading and it is often inaccurate, especially when juvenile fish have been used as input.

### FCRs mix wet and dry

The FCR is the main indicator used in aquaculture to determine the efficiency of feed. It is calculated on the basis of controlled experiments involving farmed fish by dividing the total weight of the feed consumed over the fish’s lifetime by the weight gain of fish at harvest using Eq. 2

FCR=Feed Intake/Weight Gain (2)

which describes the biological or “real” FCR; here, for example, an FCR of 1.5 means that 1.5 kg of feed was used to obtain a weight gain of 1 kg.

However, two additional factors must be considered. The first is that the FCR does not consider the reality on fish farms, which includes uneaten feed, farmed fish mortality, and escapes, which may involve thousands of fish (*48*, *54*–*59*). Thus, an “economic feed conversion ratio” can be defined which includes production losses and is therefore higher than the biological FCR. This is not considered here, but it certainly must be considered when assessing the profitability of fish farming operations.

The second factor to be considered is mainly presentational: A low FCR slightly above 1 suggests that small, reportedly inedible species (e.g., anchoveta) are transformed into large, more desirable species (e.g., salmon) nearly without losses. However, in reality, 75 to 80% of the perfectly edible fish (*60*) used as raw material for FM production are lost in the process. This is hidden by the FCR definition (Eq. 2), which uses dry weight for the feed intake (and most live fish consist of 75 to 80% water; see FishBase, www.fishbase.org/) and wet weight for the weight gain. Thus, as usually defined, the aquaculture FCR is misleading, as it suggests a production efficiency based on numbers that cannot be directly compared.

## SOURCES OF FMFO

### Reduction fisheries

**Fig. 1.**
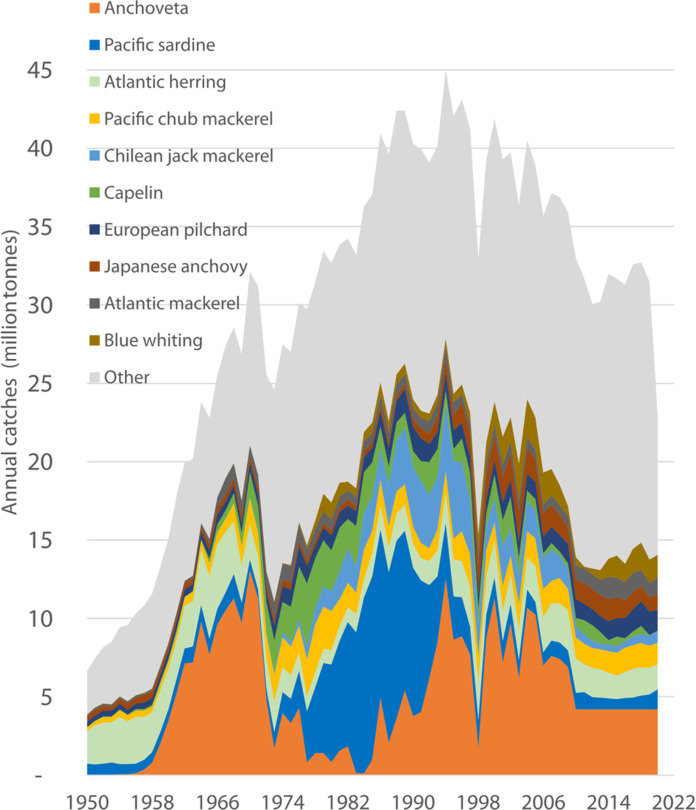
Cumulative landings of reduction fisheries 1950–2020. The graph shows the disproportionate effect of the Peruvian anchoveta fishery (bottom orange) on the shape of the curve. FAO/FISHSTAT (*100*) (www.fao.org/fishery/static/Data/Capture_2023.1.1.zip).

Twelve of the world’s top 20 fisheries (in terms of the volume of their catch) are so-called reduction fisheries (Table 2). That is, a large proportion of the global catch is ground up to produce FMFO. With an average annual catch of 23.4 million tonnes, reduction fisheries exploit some of the most important stocks of forage fish (*5*), comprising around 26% of global capture fisheries by volume (excluding algae). The largest reduction fishery, by far, is that for Peruvian anchoveta (*2*). Not long ago, this fishery accounted for 10% of all global marine catches; it is now much smaller but is still the largest single-species fishery in the world. Fluctuations in this fishery have a disproportionate impact on the global catch for reduction (up to 50% of total forage fish catch between 1958 and 2020) and, consequently, on the global supply of FMFO (Fig. 1). Two-thirds of the variability in the global supply of FM for the 1976–2016 period can be explained by variations in anchoveta landings (*61*).

**Table 2.** Top 20 fish species in the marine global catch (million tonnes) for 2010–2020 as reported by FAO/FISHTAT (*94*). Species in bold are those mainly used for reduction.

	Scientific name	Common name	Total (million tonnes)
1	***Engraulis ringens***	**Anchoveta (=Peruvian anchovy)**	**53.6**
2	*Gadus chalcogrammus*	Alaska pollock (=Walleye poll.)	36.6
3	*Katsuwonus pelamis*	Skipjack tuna	31.2
4	***Clupea harengus***	**Atlantic herring**	**19.4**
5	*Thunnus albacares*	Yellowfin tuna	15.5
6	***Scomber japonicus***	**Pacific chub mackerel**	**15.4**
7	***Sardina pilchardus***	**European pilchard (=Sardine)**	**13.8**
8	*Trichiurus lepturus*	Largehead hairtail	13.5
9	*Gadus morhua*	Atlantic cod	13.2
10	***Engraulis japonicus***	**Japanese anchovy**	**12.9**
11	***Scomber scombrus***	**Atlantic mackerel**	**11.7**
12	***Micromesistius poutassou***	**Blue whiting (=Poutassou)**	**11.7**
13	*Dosidicus gigas*	Jumbo flying squid	9.9
14	***Sardinops sagax***	**Pacific sardine**	**9.5**
15	***Sardinella longiceps***	**Indian oil sardine**	**6.2**
16	***Trachurus murphyi***	**Chilean jack mackerel**	**5.9**
17	***Sprattus sprattus***	**European sprat**	**5.8**
18	***Brevoortia patronus***	**Gulf menhaden**	**5.5**
19	*Acetes japonicus*	Akiami paste shrimp	5.4
20	*Portunus trituberculatus*	Gazami crab	5.3
			
Forage fish			171.6
Total top 20			302.2
Total global catch^*^			993.4
Average annual FF catch			23.4

*Fish, crustaceans, and mollusks only.

**Fig. 2.**
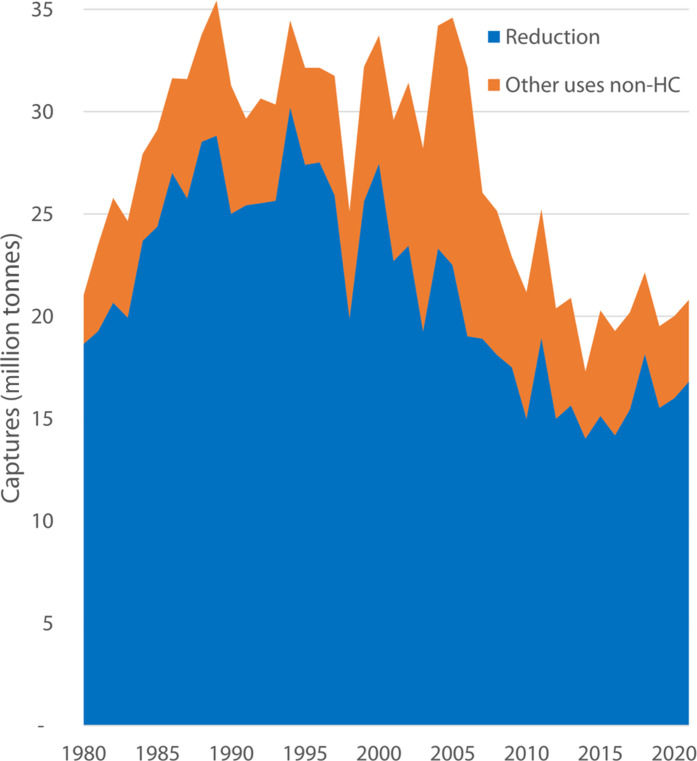
Catches for nonhuman consumption 1980–2020 (million tonnes). FAO Yearbook of Fishery and Aquaculture Statistics 1988–2019 (www.fao.org/cwp-on-fishery-statistics/handbook/tools-and-resources/list-of-fao-yearbooks-of-fishery-statistics/en/).

The catch from some fisheries, such as the anchoveta in Peru or Gulf menhaden (*Brevoortia patronus*) in the US are almost exclusively used for reduction purposes. However, for some of the other fisheries mentioned above, while the bulk of their catch is used for reduction, a fraction may be used for direct human consumption or miscellaneous nonfood purposes. The fraction used to produce FMFO varies from year to year but is not publicly reported. On the basis of reports from its member countries, the Food and Agriculture Organization (FAO) provides the only estimate of the volume of fish used to produce FMFO, showing a gradual increase from 1950, peaking at 35 million tonnes in 1989 (39% of global capture fisheries) hovering around 32 million tonnes until 2006, then decreasing and stabilizing at an average of ~21 million tonnes (23% of capture fisheries) (Fig. 2), mainly as a result of the first implementation of a harvest control rule in Peru, which notably reduced catches of anchoveta, from 8 to 10 million tonnes to less than 6 million tonnes since 2006 (*2*).

In addition to the FMFO made from reduction fisheries, an important component of China’s FMFO production comes from fisheries of what is mistakenly called “trash-fish” (*62*) or “biomass” fisheries. These involve massive amounts of miscellaneous fish and invertebrates previously discarded as by-catch, which are caught by nonselective gear, mainly bottom trawls (*63*–*66*). Over the past two decades, because of overfishing and demand for feed for aquaculture, this type of fishing has become increasingly important in Southeast Asia, involving not only Chinese vessels but also those of Vietnam, Thailand, and other countries bordering the East China Sea and the South China Sea (*67*).

There is an unknown amount of catch from these fisheries, now called feed-grade fisheries (FGF), used to produce “farm-made aquafeeds,” made by farmers for self-consumption. FAO (*2*) reports these fisheries, separately from reduction fisheries as “other non-food catches” (Fig. 2). However, since 2018, FAO reports a constant 4 million tonnes for this use, but in the East Asia region alone, around 6.4 to 8.4 million tonnes of fish were used to produce farm-made feeds in 2015 (*7*). This amount is likely to be even higher now, considering the rapid growth of aquaculture in Asia.

Much of this catch is classified in nonspecific terms such as “mixed fish,” or in FAO’s terminology as “nei” for “not elsewhere included” (*64*, *66*, *68*). A recent study (*69*) sampled feed-grade fish in 22 Chinese ports and found that almost 40% of the fish (96 species) were commercial edible fish species, 75% of which were juveniles and 40 of the species identified were categorized as overexploited. Zhang (*70*) shows that the growth in the amount of FGF used in aquaculture in China increased after 2000 and has remained around 3.5 million tonnes since 2012. Sumaila *et al.* (*66*) give a similar estimate of around 3.5 million tonnes for the past two decades. However, an accurate picture remains unclear given past and current examples of over- and underreporting of Chinese catches (*71*–*75*).

Last, an unknown amount of FMFO is also produced from the catch of Chinese fleets and processed in plants operating in West African countries—Senegal, the Gambia, Guinea Bissau, and Mauritania. These target an increasing amount of local pelagic fish, in particular sardinella species (*Sardinella aurita* and *Sardinella maderensis*), i.e., species traditionally consumed in West Africa, threatening regional food security by reducing fish availability and affordability (*67*, *72*, *76*–*80*).

### Fish processing by-products and novel materials

In response to the global decline in FMFO production from pelagic fisheries and to meet the growing demand from aquaculture, since the 1990s, between 20 and 30% of FMFO has been increasingly derived from by-products or trimmings of the seafood processing industry. According to IFFO, in 2021, almost 30% of the global FM production and 51% of the FO production were obtained from by-products. The higher percentage of FO made from by-products is mainly due to the use of a high share of farmed salmon (14.3%) and pangasius (15.3%) by-products, both having very high oil yields (*81*). Relative to FM from whole fish, however, FM from by-products has a lower protein content than that obtained from whole fish.

Suitable processing facilities and transport networks, as well as economies of scale are required for the efficient production of FMFO from by-products. Raw materials must be available in sufficient quantities, over a sufficient period to justify the required investments (*30*, *82*–*84*). At present, there are only a few locations where these conditions are met, mostly where large-scale fish processing centers are located, i.e., in China, Norway, Chile, US, and Peru. Given these limitations, FMFO from by-products may be able to cover part of the gap in FMFO supply but are unlikely to meet projected demands by 2050 (*1*, *85*).

Novel aquafeed materials, such as macroalgae, single-cell proteins (microalgae, bacteria, or yeasts), insects, or genetically modified crops, have been developed to replace either FM, FO, or both, to reduce dependency on marine ingredients (*1*, *86*–*90*). The use of these novel ingredients is growing, but their contributions to scalable and sustainable solutions are unclear. Studies on FM or FO replacement using novel feeds have yielded mixed effects on species growth and nutritional content (*1*, *91*).

Research has shown success in replacing up to a third of FO with rapeseed oil without any apparent impact on fish growth. However, there is a limit beyond which the health and nutritional quality of the fish may be compromised. Further substitution would cause docosahexaenoic acid (DHA) levels to fall below internationally recommended levels, potentially affecting consumer preferences (*74*). Without the inclusion of FO, the resulting farmed salmon would not contain a high enough level of key micronutrients, such as omega 3 fatty acids, DHA, and eicosa-pentaenoic acid (EPA), which not only are essential to the fishes’ diet but also are key elements in the marketing and branding of farmed salmon as a healthy product. FM supply, however, does not appear to impose serious limits on the quantity and efficiency of farmed salmon production (*86*).

Some of these novel ingredients are ready to be produced at a high enough scale for use in salmon feed, and some are now beginning to reach the market. So far, cost has been a barrier for their widespread production and use (*92*, *93*), at the current extreme high prices of FO (Box 1). However, this situation may change in the not distant future (*94*, *95*).

Box 1. **An Uncertain Future Supply of Fish Oil**

While increased efficiencies have been achieved in the use of FO, FO is still an essential ingredient in farmed carnivorous fish production (*67*, *74*, *86*, *155*). Thus, finding viable FO alternatives is critical as the global supply of FO from wild fish may be in decline.

The oil supply from the Peruvian anchoveta fishery—the single largest global source of this ingredient—may already be waning. Over the past 10 years, high juveniles’ catches have led to temporal closures of zones where high concentration of juveniles were reported or, in extreme cases, to shortening the fishing season (*110*, *111*, *156*). Fishing too many juveniles not only is detrimental to the sustainability of any stock but also leads to lower oil yields in reduction fisheries (*44*, *110*). In addition, the frequency of occurrence of El Niño and similar warm water events is increasing (*90*, *157*). These events decrease the typically high productivity of pelagic stocks in the Humboldt upwelling ecosystem off Peru and, consequently, the fat content of anchoveta, resulting in lower oil yields (*51*, *110*).

Furthermore, with a 2.5°C increase in mean surface water temperature that has been predicted for the year 2100 (*158*), algae may reduce the synthesis of DHA of phytoplankton by up to 28% globally, resulting in reduced levels of DHA in wild fish, which would subsequently reduce the dietary DHA available to farmed fish. Thus, depending on location, an increase in water temperature could result in ~10 to 58% loss of globally available DHA by 2100 (*49*). Cheung *et al.* (*159*) also predict a decrease in availability of DHA of 22 to 31% by 2100, but in this case, it would be as a result of projected decreases in maximum catch potential of pelagic fishes, particularly in the tropics. How these two scenarios would combine is unclear, but it is likely to imply even further decreases.

## FMFO FOR THE AQUACULTURE INDUSTRY

Almost all the global FM (87%) and FO (74%) production are currently used in aquaculture, with some of the FM used to feed pigs (7%) and poultry (1%) and some by the pet food industry (4%). For FO, an increasing amount (16% in 2021) is now used by the human nutraceutical industry and 10% for other uses (including pet food and biofuel) (*96*). Here, we look at the use of FMFO by the aquaculture industry, focusing mainly on the period from 2000 to 2020. Obtaining information on reduction fisheries has been particularly difficult. Most of the information on reduction fisheries presented here ultimately cites IFFO (*2*). However, accessing the original data has not been possible. IFFO maintains a database on production, trade, and prices for 110 countries, obtained from their member companies and various international bodies and governmental departments [FAO, Sustainable Fisheries Partnership (SFP)], producing reports only available to members (*97*). From these, they produce a Fishmeal and Fish Oil Statistical Yearbook, but to the best of our knowledge, no copies are available for consultation in any open access web site or library. We reached out to several IFFO members on multiple occasions for information but got no response. Ultimately, our main data source was FAO’s FISHSTAT database and other FAO publications (*98*–*100*), with some information obtained from presentations found on the IFFO web site (www.iffo.com/).

### Aquaculture and the use of FMFO

Fed aquaculture is the largest and fastest growing component of the aquaculture sector (excluding algae), and its production has tripled in the past 20 years (*2*). Excluding algae, global aquaculture production in 2020 was estimated at 87.5 million tonnes (*99*), with China being responsible for almost two-thirds of the global aquaculture production (*101*). In 2020, of the 70 million tonnes of aquaculture production in China, 20 million tonnes consisted of algae (30%); of the remaining 50 million tonnes, ~40% (~20 million tonnes), were composed of finfish, crustaceans, and other carnivorous species that require animal feed (Table 3). This has resulted in China consuming 60% of the world’s FMFO production, up from about 5% in 1990 (*29*, *63*, *72*).

**Table 3.** Marine and freshwater aquaculture production, divided by fed and nonfed groups (tonnes × 1000) for China and the World for 2020 FAO/FISHTAT (*93*).

			Production (tonnes × 1000)		
Habitat	Fed	Major group	China	World	%
Freshwater	n	Mollusks	187	193	0.4
		Finfish	17,255	27,877	52.2
		Invertebrates*	56	56	0.1
	y	Other**	518	537	1.0
		Crustaceans	4,258	4,477	8.4
		Finfish	8,609	20,290	38.0
		**Total freshwater**	30,883	53,430	
				
Marine	n	Mollusks	14,801	17,549	51.5
		Finfish		105	0.3
		Invertebrates	412	469	1.4
	y	Crustaceans	1,775	6,760	19.8
		Finfish	1,750	9,190	27.0
		**Total marine**	**18,738**	**34,072**	
		**Total animals**	**49,621**	**87,503**	
					
	Algae	Freshwater	63	64	
		Marine	20,800	35,013	
		**Total algae**	**20,863**	**35,078**	
		**Toatal aquaculture production**	**70,484**	**122,580**	

*Nonmollusks or crustaceans.

**Frogs and turtles

Although most of the aquaculture production in China requires no feed (Table 3), the fraction that does (40% or 19 million tonnes) generates the massive demand for feed that makes them the top global FM consumer. Also, China’s increasing aquaculture production in offshore environments is a worrying trend. Apart from greater fuel costs, operations in high-energy offshore environments include the use of reinforced and submersible cage structures, high levels of automation, and large sizes to capture economies of scale. This makes offshore aquaculture a relatively high-cost endeavor, therefore requiring industrial-scale focus on high market value species to offset production costs. Projects currently proposed in Chinese waters are scheduled to produce salmon, large yellow croaker (*Larimichthys crocea*, a fish highly appreciated in China), or a mix of luxury species that include Japanese seabass, puffer fish, tuna, and yellowtail amberjack (*Seriola lalandi*)—all high value marine carnivorous finfish species requiring high levels of FMFO (*92*, *102*, *103*).

China is not, however the largest consumer of FO. The primary global FO importers are Norway and Chile, which mainly produce Atlantic salmon, a species that relies heavily on FO in its feed (*17*). Salmon farming has grown substantially since it started in the 1960s, and today, ~70% of salmon consumed worldwide is farmed. In 2020, around 2.7 million tonnes of farmed salmonids was produced (*100*) with Atlantic salmon alone accounting for 32% of all marine finfish aquaculture production and 60% of fish oil usage (*4*, *46*, *104*). As this consumption continues to grow, where will the additional FMFO come from? It is clear that the current supply by wild fish is not sufficient to cover the global demand.

**Fig. 3.**
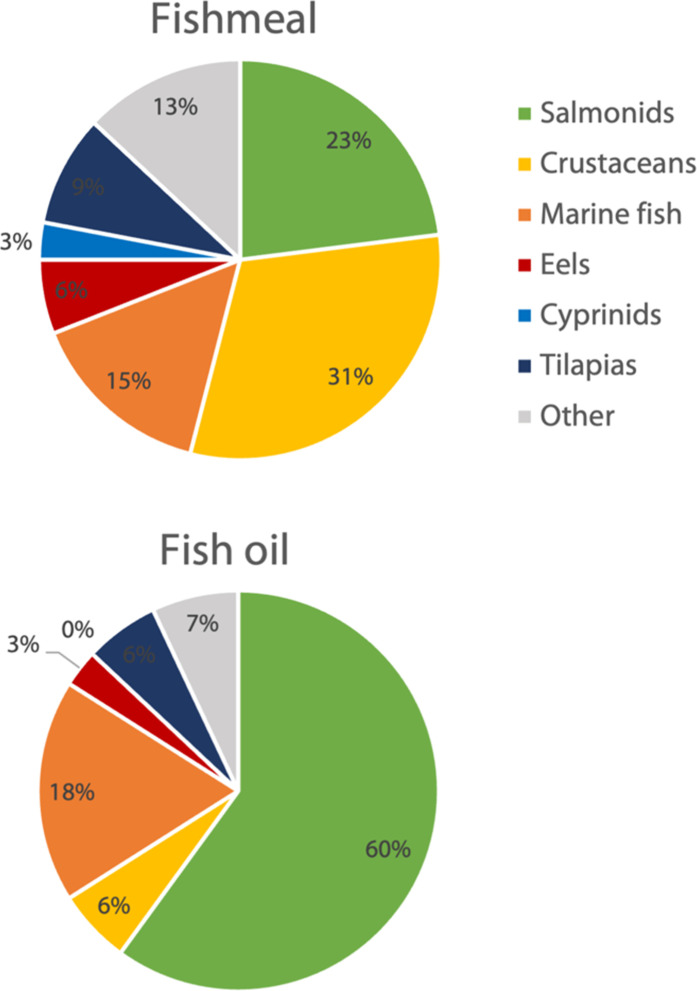
Use of fishmeal and fish oil in aquaculture in 2016. Adapted from Seafish (*4*), citing the IFFO Fishmeal and Fish oil Statistical Yearbook 2017.

Despite comprising just 14% of global aquaculture production in weight, most FM (75%) and FO (84%) used in aquaculture in 2016 were allocated to high-value species (Fig. 3) such as salmonids, eels, other marine carnivore fish and to shrimp (*4*). Why are these species, especially salmon, so valuable that they consume such an important portion of these vital resources? The answer lies in trade.

Most aquaculture production does not enter the global market. Global aquaculture trade is largely limited to a select few species, with Atlantic salmon and white-leg shrimp (*Pennaeus vannamei*) accounting for ~6 million tonnes, which is 9% of global seafood production but 32% of the value of internationally traded seafood (*98*). These species have become global commodities for which future markets have been established; large international companies are better able to exploit economies of scale (*105*, *106*). Also among the most valuable “seafood” trade products are FM and FO, and even trimmings (fish waste). Nowadays, farmed salmon alone, shrimp, FM, and FO account for 35% of the global seafood trade value and 26% of its weight (*98*).

Future production potential for fed mariculture species—salmon and similarly high value carnivore species—is tightly coupled to feed availability and access. Costello *et al.* (*107*) modeled the production potential for fed marine finfish considering economic factors (i.e., costs and profitability) and future feed scenarios in addition to environmental suitability. They found that finfish production in general only becomes economically viable when the production price equals 5000 USD per tonne that in 2019 was below the price for Atlantic salmon at 7000 USD per tonne.

**Fig. 4.**
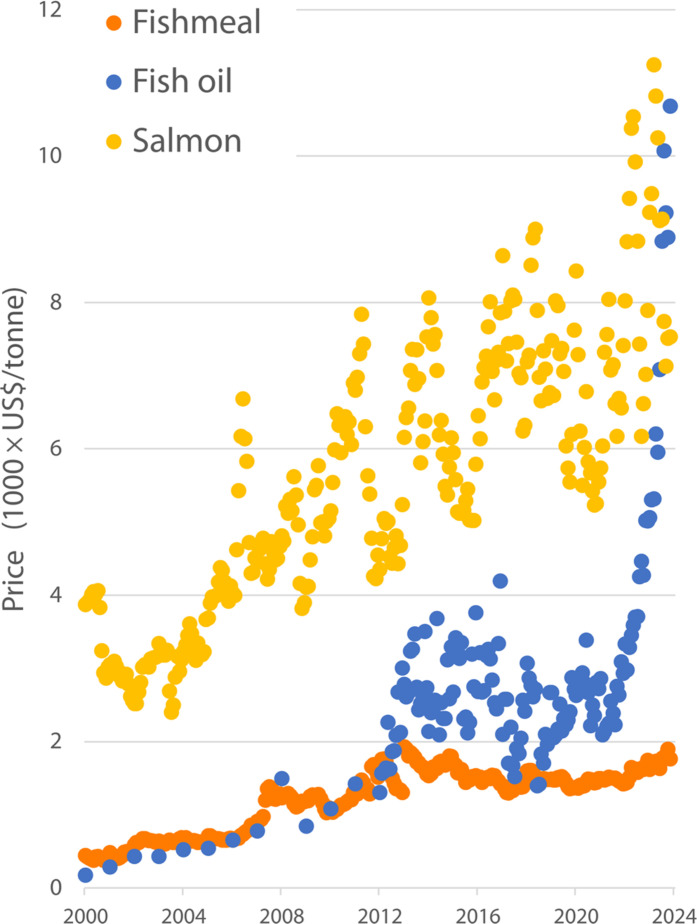
Variations in global prices of fishmeal, fish oil, and salmon 2000–2024 (USD per tonne). Sources: fishmeal and salmon: www.indexmundi.com; fish oil: Banco Central de Reserva del Peru (https://estadisticas.bcrp.gob.pe/estadisticas/series/mensuales/resultados/PN38769BM/html).

That analysis was conducted when FO prices were below 3000 USD per tonne, which is where they were throughout the 2000s and 2010s (Fig. 4). However, FO prices then began to steadily rise, surpassing 11,000 USD per tonne in January 2024 (*108*). Concurrent with the rise in the price of FO, the global price of salmon began to fluctuate wildly, also passing 10,000 USD per tonne in September 2023 (*109*).

The main reason for the FO price increase was the global shortage resulting from the low oil yield in the Peruvian anchoveta fishery resulting from high juvenile catches since 2021 (*44*, *110*). In addition, because of an El Niño event, the first fishing season of 2023 was canceled, and a quota lower than usual was approved for the second season, when El Niño conditions were predicted to get worse (*111*–*115*). This means that the supply of FO from Peru in the upcoming months is likely to be very limited or nonexistent, and prices probably will remain high (*116*–*118*), with important consequences on the use of FO in the future.

**Fig. 5.**
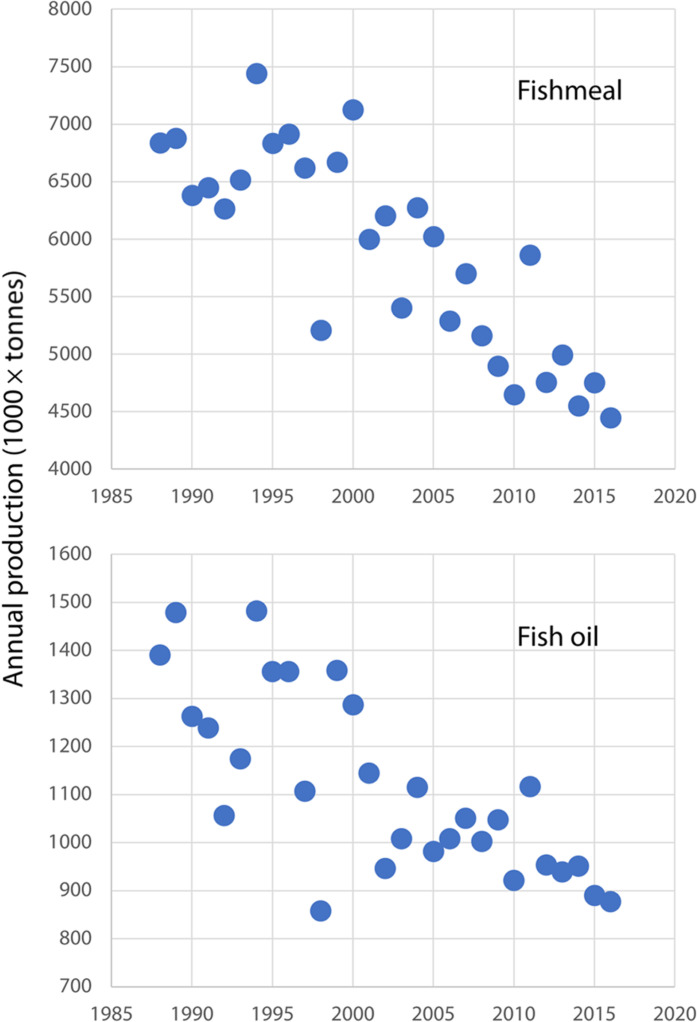
Historical trend of world production of FMFO (million tonnes) 1963–2016. Data obtained by digitizing figure 10 of Tacon, Hasan, and Metian (*37*) (www.fao.org/3/ba0002e/ba0002e.pdf).

Global supplies of FMFO from pelagic fisheries have been declining for the past two decades (Fig. 5) (*2*, *30*–*32*, *86*), while the global production from by-products has remained roughly constant since 1990 (*2*). Already, 51% of the global FO supply is obtained from by-products from the seafood processing industries (*81*). Some more FO may be obtained from these processes, but projections for this supply apparently will not be sufficient to cover the big gap that will open, given the salmon industry’s growth projections of around 4 to 5% (*1*, *85*, *119*, *120*). The impending shortage of FO explains the growing Norwegian and Japanese fisheries for Antarctic krill (*Euphausia superba*) (*121*). Also growing is the Norwegian interest in developing efficient methods for catching small mesopelagic fish such as lanternfish (family Myctophidae). Small mesopelagic fish, contrary to krill, rarely form dense schools and therefore, despite their overall abundance, have failed to sustain large-scale fisheries (*122*).

Last, China is the second largest economy in the world (or the largest but for some measures) and is expected soon to graduate from “middle income” into “high income” under the World Bank country classification system (*123*). With greater affluence, Chinese consumers are increasing their consumption of aquatic products, and this consumption is projected to continue to grow and require a minimum of 6 million tonnes of additional seafood to cover the demand to 2030 (*124*). In addition, Chinese middle and upper classes are not only eating more seafood per capita, they are also consuming more high-value species, which means that China’s and, therefore, global demand for FMFO will be even greater in the future.

## DISCUSSION

### The tale of the FIFO metric

Previous discussions about the quantity of wild fish required to produce farmed fish have used FIFO as a primary metric (*11*, *14*, *15*, *17*, *18*, *21*). To show a decreasing dependence on wild fish, a variety of formulas to calculate the FIFO metric have been proposed. These are largely reformulations of the original Tacon *et al.* (*27*) equation but later iterations, like the economic (e)FIFO of Kok *et al.* (*25*), allocates economic value to the different ingedients included in aquafeeds. All of these variations, however, likewise confirm that the amounts of FM and FO included in aquaculture feeds has drastically decreased over time, especially when by-products are also included.

To reach this optimistic conclusion, these exercises often include a table comparing species to produce an average FIFO, usually well below 1. They give average FIFO values that include carnivores, omnivores, and herbivores, thus masking the much higher FIFO values for carnivores. For example, Table 4 taken from table 16.2 of Jackson and Shepherd (*27*) shows their calculation of the use of FO in farmed seafood production, where they average salmon with a FIFO value of 4.08 with species that barely require any FO and therefore have FIFO values lower than 1 and most of them close or equal to zero (Table 4).

**Table 4.** An example of IFFO's calculation of the use of FO in farmed production. The average value is then taken as evidence for the reduction of the FIFO for aquaculture over time and is also used to counter the argument that it does not take 5 kg of wild fish to produce 1 kg of salmon, while the FIFO value for salmon reported on this table is still high. This is an adaptation of an original work by the Organisation for Economic Co-operation and Development (OECD). The opinions expressed and arguments used in this adaptation are the sole responsibility of the author(s) of the adaptation and should not be reported as representing the official views of the OECD or of its Member countries. [Reproduction of table 16.2 of Jackson and Shepherd (*27*).]

	Fish oil	Raw material	Whole fish	Farmed production	FIFO
Human consumption	126	2,689	2,017	–	–
Other uses	110	2,340	1,755	–	–
Crustaceans	28	589	442	4,673	0.09
Marine fish	115	2,455	1,841	2,337	0.79
Salmon and trout	604	12,857	9,642	2,365	**4.08**
Eels	15	320	240	244	0.98
Cyrprinids	1	24	18	13,037	
Tilapias	18	376	282	2,737	0.10
Other freshwater	15	313	235	2,102	0.11
Aquaculture subtotal	795	16,934	12,700	27,495	**0.46**
Total	1,032	21,964	16,472		

Furthermore, on the IFFO web site (www.iffo.com/), one finds the claim that the average FIFO for fed aquaculture has decreased to 0.19. This average FIFO value would mean that 0.19 kg of wild fish is needed to produce a kg of farmed fish. This is then taken by the authors to conclude that “for every 1 kg of wild fish used 5 kg of farmed fish are produced”. This is not quite true as the feed used to produce the farmed fish has a minimal FMFO inclusion [less than 10% for farmed Atlantic salmon (*96*)] and therefore, the farmed fish growth is largely the product of the other nonfish ingredients that make up most of the feed’s composition. This is particularly true since the global scarcity; growing demand and higher prices have led to ever lower wild fish FMFO inclusion in feeds and a more selective use of these ingredients in grower diets (*29*, *36*, *96*).

It is further claimed in the same web site that in 2020 the FIFO for salmonids was below 1.0—without any supporting data—and thus conclude that “the salmonid feed industry supports the production of more farmed fish than it uses as feed fish” and that “The marine ingredients industry supports the production of a significantly greater volume of protein for humanity than would be supplied merely through the direct consumption of the fish used as raw material in the production process” (*26*). Again, the contribution of by-products and other nonfish components of feed are not mentioned.

Also, setting aside the questionable practice of averaging FIFO values for herbivores and carnivores, the fact remains that these calculations were done assuming constant FM or FO yields, inclusion rates and FCRs, which, as noted earlier, can vary widely. IFFO uses fixed FM (22.5%) and FO (5%) yields in their FIFO calculations (*21*, *26*, *74*, *125*), despite evidence that FO yields especially can vary by an order of magnitude even within a species and across years and locations (*42*, *44*, *51*).

### How are FMFO being used?

Given the impact on prices of the recent drop in production in the Peruvian anchoveta fishery, it appears that the current global supply of FO is insufficient to cover the existing demand. At present, all current supplies of FO, both from whole fish and from by-products, are being used by the different industries that require them—but primarily by the aquaculture industry—and the search for alternatives (e.g., algae and insects) is underway (*126*). If FO prices were to remain at the extreme level reached in 2023, some of these alternatives may lastly become economically viable and come online, particularly for the production of high-value species such as salmon.

The growing use of FMFO produced from by-products is often suggested as evidence of a decreasing demand for wild fish derived FMFO (*33*, *74*, *127*). Another interpretation of that same trend is that the use of FMFO from by-products reflects the ever-increasing—probably unsatisfied—demand of the aquaculture industry for marine ingredients. They supplement rather than replace FMFO from whole fish and help reduce costs. Nowadays, more than half the FO (53%) and 34% of the FM used in aquaculture come from by-products (Fig. 4) (*96*). Cao *et al.* (*82*) estimated that by-products could meet around half, and potentially two-thirds, of China’s current demand for FM in aquafeeds. Although global aquafeed production has quadrupled in the past two decades, the global use of FM has not seen a proportional increase. The absolute amount of FM has remained relatively constant, but inclusion rates have declined by about a third (*29*, *31*, *36*, *37*). The aquaculture industry is fast shifting to terrestrial crop-based feed ingredients, such as soy, to replace wild fish FMFO and reduce production costs (*36*).

Nowadays, the use of FO in low-value omnivores is almost nil; most is used to feed the higher-value, mostly carnivorous species (Fig. 4). At the current extreme FO prices only the very lucrative Atlantic salmon industry (as well as the Omega-3 pill industry and more recently, the high-end pet food industry), may be able to afford the use of FO. As prices continue to increase, eventually, all the FO used in aquaculture may end up being used to feed mainly salmon and other high-value carnivore fish.

### Are FMFO providing food security?

Aquaculture has been touted as the fastest growing animal food production system with great potential to feed and nourish the world’s growing population (*2*, *91*, *128*, *129*). These claims have been contested in a number of ways (*92*, *130*–*135*) but perhaps, one of the most controversial aspects has always been that of its real contribution to world food security and nutrition (*3*, *30*, *67*, *136*–*139*). While the aquaculture industry regularly uses the narrative of food security, their top products, salmon and shrimp, are prized not for their nutritional value but for their export value (*134*). In the case of fed aquaculture, and salmon in particular, because of its high dependence on FO, it is actually driving the reduction industry to take food away from people.

The Chinese distant water fleet and processing plants operating in West African countries—Senegal, the Gambia, Guinea Bissau, and Mauritania—exploit local pelagic fish to produce FMFO. Fishmeal factories are using an increasing amount of fish, in particular, sardinella species (*S. aurita* and *S. maderensis*), i.e., species traditionally consumed in West Africa, threatening regional food security by reducing fish availability and affordability (*67*, *72*, *76*–*80*).

The scarcity of FO throughout 2023 triggered a rush for finding quick new sources of small pelagic fish. For example, companies in Norway purchased relatively expensive fish normally used for human consumption—mackerel and sardines—to produce FO, when “high fish oil prices made the business profitable” (*116*). Even aquaculture itself is becoming a major source of FMFO through the reuse of processing waste (*81*). If prices remain as high as they currently are, there may come a time when FMFO produced from by-products or trimmings of cheaper species such as catfish and tilapia may become more valuable than the fish parts that would typically be used to feed people. If this were to happen, then, these fish could easily become yet another source of FMFO to feed the ever-growing demand of the fed-aquaculture industry, further affecting global food security.

Millions can and do eat and enjoy these fish in the countries where they are caught (*60*, *140*). It has been the reduction industry—IFFO in particular—that has insistently tried to justify the continued use of most of the global catch of forage fish to produce animal feeds. These fish are the most affordable and nutritious wild fish in most countries, particularly in low-income African countries, such as Uganda and Guinea (*141*). IFFO (*142*) claims aquaculture is the most effective way to use the nutrients found in small pelagic fish, but there is evidence that this is not true. A recent study examined micronutrient flows from feed to farmed fish and showed that less than half of the essential dietary minerals and fatty acids in wild fish were retained in farmed salmon (*135*). Moreover, the full nutritional role of ingesting whole small pelagic fish is extensively discussed by Bavinck *et al.* (*143*).

### Is it possible to redirect the use of FMFO to provide food for humans?

Aquafeeds must satisfy the nutritional prerequisites of the target animal without a requirement for any specific ingredient. They must provide the correct combination of nutrients to fulfill the metabolic needs of the species in question and provide the taste and nutrition that consumers look for. That is why it has been possible to significantly reduce, and in some cases eliminate, the use of FMFO in feeds for most species, except for those like salmon that still require FO to maintain the taste and nutrition profile people expect.

A justification for the continued use of small pelagic fish for reduction has been that “markets for small pelagic fish direct human consumption are not and never have been well established” (*28*). This is not true, as any visits to real fish markets in Southeast Asia or West Africa will confirm (*144*, *145*). Moreover, new markets for human consumption for several reduction species, like capelin (*Mallotus villosus*), Atlantic (*Clupea harengus*), and Pacific herring (*Clupea pallasii*), and even anchoveta have been found (*60*, *140*), and it is possible to change consumer food preferences to develop new markets (*146*). For example, monkfish (*Lophius piscatorius*) was considered a fish for the poor and rejected because of its appearance—now it is sought after and expensive, and the case is similar for lobster (*147*).

There is no assurance that decreasing reduction fisheries will result in increased food security and nutrition though. Some successful campaigns to increase consumption of small pelagic, oily fish such as anchoveta, sardines, and mackerel have targeted affluent consumers, aiming to reposition the fish as high-end, gourmet products, to make people aspire to eat the fish usually seen as food only fit for the poor (*60*, *140*, *146*). These campaigns could result in increased consumption by the richer target audiences, without achieving improved food security and nutrition. However, nowadays, most of the FMFO produced worldwide is sourced from countries in the Global South, and over the past decade, Northwest Africa has become a key sourcing region for the production of FMFO.

The industrial production of FMFO is driving up the price of fish and depleting marine resources in traditional fishing areas, reducing the availability of fish for human consumption. Just in Senegal, between 2009 and 2018, fish consumption declined by 50% (*148*–*150*). Similarly, in Southeast Asia (*65*, *68*, *134*, *151*) and India (*152*), what has been called “trash” fish but were formerly available to people, are taken by the reduction industry or directly used to feed farmed fish or shrimp. Also, pollution and noxious stench coming from FMFO factories in the Global south affect the neighboring communities’ quality of life. Thus, decreasing or eliminating reduction fisheries from West Africa and/or Southeast Asia and the Indian Ocean would most likely result in improved food security and nutrition for their impoverished populations, with the added benefit of the improved recovery potential and resilience for the already overexploited ecosystems from where the fish are taken.

Yet, the economic trade-offs associated with restricting the use of forage fish in the reduction industry would probably make it very difficult to achieve change. We have seen the immediate and huge impact that closing the Peruvian anchoveta fishery has had on global prices of FO. At such prices, it would be impossible to compete for access for the same fish to produce food, especially if the food needs to be widely affordable. At the current prices, even salmon may soon become inaccessible to many that can afford to buy it now.

### New opportunities

Climate change makes the possibility of business as usual highly unlikely. The latest predictions for the impacts of climate change on the Peruvian anchoveta stock—the main global source of FMFO—are worrying. The optimistic scenario is a 14% per decade reduction of biomass until mid-21st century, followed by a collapse and late recovery by the end of the 21st century, with no changes in spatial distribution of the population. The pessimistic scenario is a reduction of biomass of 22% per decade, with collapses and near extinction by 2060, with a spatial displacement of the population to the south and to more coastal areas (*153*). So, the closure of the anchoveta fishery in 2023 could be but a prelude for the catastrophes that are likely follow.

In their paper projecting global mariculture production and adaptation pathways under climate change, Oyinlola *et al.* (*154*) seem to foretell the situation currently happening as a result of the FO shortage: “Revenue from mariculture is highly reliant on demand elasticity, which is responsive to change in commodity pricing. Climate change could cause a decline in the supply of seafood production from mariculture, which might impact the price of seafood and consequently affect mariculture production.”

Oyinlola *et al.* (*154*) projected the dependence of mariculture production on FM production around a crude protein index—basically equivalent to a FM inclusion rate. They did not include FO in any of their calculations. Still, they projected a decrease in global FM production by the mid and end of the 21st century, causing developed countries with mariculture such as Norway, Japan, and Australia, where the mariculture of carnivores is highly developed, to face reductions in their mariculture production, with negative consequences on their seafood trade and associated employment. Oyinlola *et al.* (*154*) also found that the variations in regional impacts in mariculture production potential were strongly dependent on the type of species farmed and their associated FM requirement. Finfish production would be considerably more affected than mollusks’ (nonfed species) as a result of climate-related declines in the forage fish species used to produce FMFO.

Also, when comparing varying degrees of dependence on FM (using feeds with different percentages of FM substitution with nonfish alternatives), Oyinlola *et al.* (*154*) found that farming using high fish-dependent feeds was likely to be most affected. They thus proposed developing low FM-dependent feeds as a practical and effective adaptation strategy to climate impacts, stating that “The decrease in FMFO supplies as a result of climate change may accelerate and/or force adaptation responses by the mariculture industry, such as limiting the use of FMFO as a feed ingredient” and “It may also stimulate investment into low FMFO-dependent aquafeed development.”

Had Oyinlola *et al.* (*154*) included FO in their calculations, given the much greater supply limitations on FO relative to FM, it is very likely that the projected impacts of climate change on mariculture production for fed finfish with high-fish feed dependencies would have been much greater. We are now already seeing how quickly and markedly FO prices increase after a short-term FO production shortage, with salmon prices following closely. We have not yet seen the impacts on consumption and/or future production. However, if prices remain as high, perhaps only the large producers operating at scale can absorb those FO costs.

Reducing or eliminating the use of forage fish in aquaculture will only make aquaculture more resilient to climate change. Doing so would also encourage the many novel feed alternatives that are in development to come to fruition with the attractive potential investments these represent. In addition, there are a myriad social, economic, and ecological benefits of leaving more forage fish in the ocean and/or using forage fish to instead directly improve human nutrition and livelihoods.
